# Consumption of Food Components of the Mediterranean Diet Decreases the Risk of Breast Cancer in the Makkah Region, Saudi Arabia: A Case-Control Study

**DOI:** 10.3389/fnut.2022.863029

**Published:** 2022-04-18

**Authors:** Firas S. Azzeh, Deena M. Hasanain, Alaa H. Qadhi, Khloud J. Ghafouri, Wedad F. Azhar, Mazen M. Ghaith, Abdullah F. Aldairi, Hussain A. Almasmoum, Hamza M. Assaggaf, Maha H. Alhussain, Ahmad A. Alghamdi, Mahmoud M. Habibullah, Waleed M. Bawazir, Sofyan S. Maghaydah, Maysoun S. Qutob, Awfa Y. Alazzeh

**Affiliations:** ^1^Clinical Nutrition Department, Faculty of Applied Medical Sciences, Umm Al-Qura University, Makkah, Saudi Arabia; ^2^Clinical Nutrition and Dietetics Department, Faculty of Pharmacy, Applied Science Private University, Amman, Jordan; ^3^Clinical Nutrition Department, King Abdullah Medical City in Holy Capital (KAMC-HC), Makkah, Saudi Arabia; ^4^Department of Laboratory Medicine, Faculty of Applied Medical Sciences, Umm Al-Qura University, Makkah, Saudi Arabia; ^5^Department of Food Science and Nutrition, College of Food and Agriculture Sciences, King Saud University, Riyadh, Saudi Arabia; ^6^Department of Clinical Laboratories Sciences, College of Applied Medical Sciences, Taif University, Taif, Saudi Arabia; ^7^Medical Laboratory Technology Department, Faculty of Applied Medical Sciences, Jazan University, Jazan, Saudi Arabia; ^8^Medical Research Center, Jazan University, Jazan, Saudi Arabia; ^9^Department of Medical Laboratory Technology, Faculty of Applied Medical Sciences, King Abdulaziz University, Jeddah, Saudi Arabia; ^10^Hematology Research Unit, King Fahd Medical Research Center, King Abdulaziz University, Jeddah, Saudi Arabia; ^11^Department of Clinical Nutrition, College of Applied Medical Sciences, University of Ha'il, Ha'il, Saudi Arabia

**Keywords:** breast cancer, postmenopausal, Mediterranean diet, dietary habits, nutrition

## Abstract

**Background:**

Breast cancer is one of the leading causes of death worldwide, it affects both men and women. In Saudi Arabia, breast cancer has been the most prevalent type of cancer in women, for the past few years. Dietary habits and cultural beliefs vary according to region, and further studies are required to demonstrate the relationship between these dietary habits and cultural beliefs and the risk of developing breast cancer. This study is aimed to discover the relationship between preventive dietary factors of the Mediterranean diet and rates of breast cancer among postmenopausal women in the Makkah region of Saudi Arabia.

**Methods:**

A case-control study was conducted in King Abdulla Medical City Hospital, Makkah, Saudi Arabia and included 432 Saudi female participants: 218 in the control group and 214 breast cancer patients. All participants were postmenopausal, around the same age, and all were ethnically Arab Saudis. Data were obtained using a self-administered validated questionnaire.

**Results:**

Study results showed that a diet that includes 1–2 servings of legumes weekly, 1–5 servings of fish weekly, 1–5 servings of dairy products daily, 3–5 servings of fruits and vegetables daily, and more than one cup of black tea and coffee per day significantly (*p* < 0.05) reduces the risk of breast cancer.

**Conclusion:**

This study demonstrates that consuming a Mediterranean diet, which includes legumes, fish, fruits and vegetables, black tea, coffee, and low intake of dairy products, works as a preventive factor against breast cancer in postmenopausal females from the Makkah region.

## Introduction

Breast cancer is one of the leading causes of death worldwide, with 2.3 million newly diagnosed women in 2020 (1). In Saudi Arabia, breast cancer is the second most common type of cancer, after rectal cancer, and is responsible for 3,629 (14.8%) new cases diagnosed in both genders. Breast cancer affects women more than men and represents 29.7% of newly diagnosed cancer cases in women (2). In 2014, the tumor registry in King Faisal Specialist Hospital and Research Center reported that breast cancer represented the highest percentage of cancer cases at 11.7% (3). The rapid increase of cases among different age groups, during the past few years, makes breast cancer one of the most critical topics to study in the medical field. About 5–10% of cancer cases are caused by genetic factors, while 90–95% cases are related to environmental factors and unhealthy lifestyle elements, such as diet, obesity, and alcohol consumption (4).

In addition to a high intake of olive oil, the Mediterranean diet focuses on a plant-based consuming pattern with high fiber intake from fruits and vegetables, legumes, and cereals and high omega-3 from fish and kinds of seafood. Furthermore, moderate intake of dairy products and low intake of red meat and poultry are also recommended in the Mediterranean diet (5). A recent case-control study showed that following the Mediterranean diet rich in olive oil, fish, fruits, and vegetables reduces the risk of breast cancer in pre- and postmenopausal women (5).

Several risk factors contribute to increased risk of breast cancer, which include alcohol, obesity, a sedentary lifestyle, exogenous estrogen and progesterone, menarche at an early age (<12 years old), previous surgeries or biopsies, previous mammography screenings, and never giving birth or breastfeeding are all risk factors for breast cancer (6, 7). However, a healthy diet and lifestyle have been shown to reduce the incidence of breast cancer. Therefore, verifying the risk factors for breast cancer is of vital importance. Several studies have assessed the influence of lifestyle and nutrition on breast cancer (8, 9). Fruits, vegetables, dairy products, and olive oil were found to be preventive against breast cancer (10). A case-control study in Iran showed that a high level of vitamin D in the fourth quartile of serum 25(OH)D decreased the risk of breast cancer (11). However, that study showed that high consumption of meat and fat increased the risk of breast cancer (12).

Regional differences in diet and lifestyle habits play a role in breast cancer development; women in Saudi Arabia have different dietary habits depending on the region they live in and their cultural beliefs. Further studies are required to investigate the effect of dietary habits on breast cancer in different cultures. Additionally, women after menopause could be at higher risk of breast cancer than premenopausal women (6). Therefore, this research is aimed to explore the causation and preventive factors of breast cancer in postmenopausal women from the Makkah region, particularly the impact of consuming a Mediterranean-based diet on the prevalence of breast cancer.

## Materials and Methods

### Study Design and Setting

This case-control study took place in King Abdullah Medical City Hospital in the Makkah region of Saudi Arabia from June 2014 to November 2016. This hospital is the biggest in the region and the only center that provides screening and treatment of cancer locally.

### Participants

**Figure 1:**
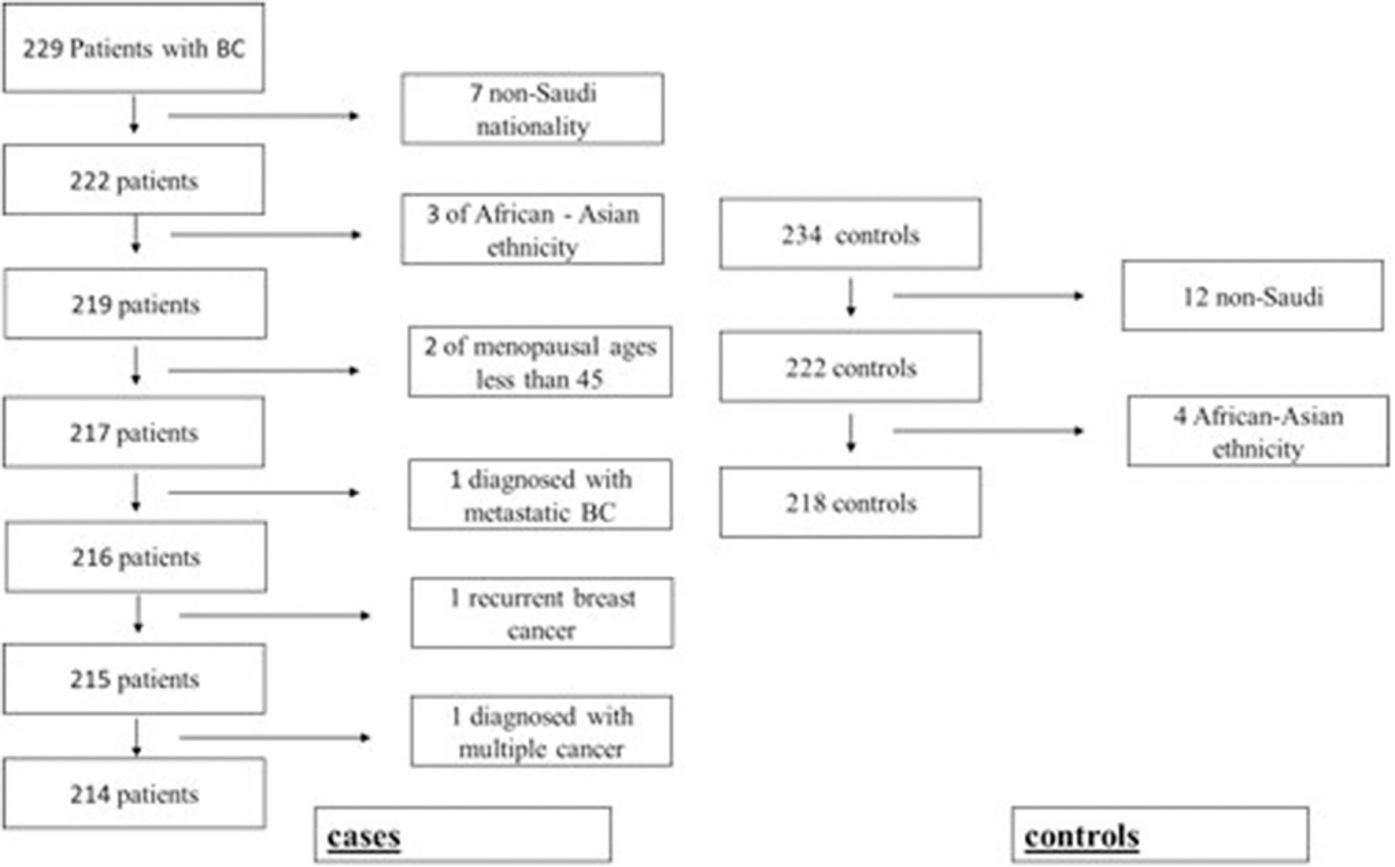
Flowchart for recruiting cancer patients and control group participants.

A total of 432 female participants were recruited (214 cancer cases and 218 controls). Only Saudi postmenopausal women who were newly diagnosed with breast cancer and aged above 45 years old were included. A postmenopausal woman is defined as any woman who no longer has her periods for at least a year. The mean ages of the case and control groups were 57 ± 7.3 years old and 56.9 ± 8.6 years old, respectively, which was not significantly (*p* > 0.05) different. Additionally, no significant differences (*p* > 0.05) were found in caloric intake (1,916 Kcal ± 437.7 and 1,837 Kcal ± 392.4, respectively) between the case and control groups. Diagnoses of breast cancer patients were confirmed by biopsy by the oncologist in the same hospital. To control the racial factors, only ethnically Arab women were invited to this study. The control group was healthy women without any acute or chronic disease and they were chosen from the hospital workers and patients' families or friends. They were selected from the same region and age range as the cases with a single year of age in the match and without any acute or chronic disease. Figure 1 is a flowchart that shows inclusion and exclusion criteria for the recruitment of participants. The response rates for the case and control groups were 93.4 and 93.2%, respectively.

The study data were collected after the investigation was approved by the Institutional Review Board of Umm Al-Qura University (approval number AMSEC-2-20-5-2014), following the Declaration of Helsinki rules. Eligible women read and signed the consent form before starting the data collection.

### Data Collection

Data for this study were collected using convenience sampling and face-to-face interviews. Participants were asked to fill out a questionnaire, supervised by a registered dietitian. The questionnaire recorded personal information and questions about dietary habits; a validated dietary questionnaire from the 2017 work of Azzeh et al. (13) was used. The questionnaire included questions about daily intake of foods, such as fruits and vegetables, meat and processed meat, poultry, dairy products, and beverages, such as coffee and black tea (*Camellia sinensis*). In the Saudi community, black tea is generally offered in an 80-ml cup, while coffee is offered in a 50-ml standard cup. Other food categories that include fish and seafood, olive oil, green leafy vegetables, and legumes were recorded weekly. Daily bread consumption and preferences for either white or whole wheat bread were also assessed. All participants were taught about the serving size for each food item prior to starting the questionnaire. The registered dietitian measured the height and weight of the participants using the Detecto physician scale, available at the hospital (Detecto, Webb City, Missouri, USA). Body mass index (BMI) was calculated by dividing weight in kilograms by height in meters squared.

### Statistical Analysis

Statistical tests in this study were completed using IBM SPSS Statistics for Windows, version 20.0 (IBM Corp., Armonk, NY, USA); a value of *p* < 0.05 was set for the significant differences. A suitable test was used to decide the value of *p* for each parameter. The data were arranged using a case-control status to verify the differences between the cases and the controls. To assess the risk factors related to breast cancer, the odds ratio (OR), 95% confidence intervals (95% CI), and β-coefficient were determined. All variables were adjusted according to the confounders described in Alsolami et al.'s article (14), which were as follows: BMI (continuous), total caloric intake (continuous), employment, income, education level, family size, smoking, physical activity, cancer background, menstruation start, and contraceptives usage.

## Results

Results of this study showed that the employment rate in the control group (41.2%) was higher (*p* < 0.001) than the case group (13%; Table 1). The percentage of participants in the control group who had a low monthly income of <5,000 SR (4.8%) was lower (*p* < 0.001) than that of the case group (21.8%). The literacy percentage in the control group (0.5%) was lower (*p* < 0.001) than that of the case group (7.4%). The case group had a noticeably larger (*p* < 0.001) family size as compared to the control group. Menarche at an early age (<10 years old) was remarkably higher (*p* < 0.001) in the case group as compared to the control group. The consumption of hormonal contraceptives was higher (*p* < 0.001) in the case group as compared to the control group (21.8 vs. 12.7%, respectively). No differences (*p* > 0.05) were shown regarding marital status, family history of breast cancer, age of menopause, and breastfeeding duration in either control group or case group. The complete details for the baseline characteristics of the participants were published previously in Alsolami et al.'s research (14).

**Table 1:** Baseline characteristics of the participants (*n* = 432 total).

**Parameter**	**Control**	**Case**	***P*-value**
Body Mass Index (BMI) (kg/m^2^)	27.7 ± 6.3	35.4 ± 10	<0.001
**Employment**			
Yes No	178 (41.2%) 40 (9.3%)	56 (13%) 158 (36.5%)	<0.001
**Family income**			
<5,000 SR^*^ 5,000–10,000 SR 10,000–2,0000 SR >20,000 SR	21 (4.8%) 90 (20.8%) 80 (18.5%) 27 (6.2%)	94 (21.8%) 88 (20.4%) 24 (5.6%) 8 (1.9%)	<0.001
**Education**			
Illiterate Primary Intermediate/secondary Postsecondary	2 (0.5%) 3 (0.7%) 23 (5.3%) 190 (44%)	32 (7.4%) 96 (22.2%) 38 (8.8%) 48 (11.1%)	<0.001
**Family size**			
5 or less 6 or more	115 (26.6%) 103 (23.8%)	40 (9.3%) 174 (40.3%)	<0.001
**Smoking**			
Yes No	3 (0.7%) 215 (49.8%)	38 (8.8%) 176 (40.7%)	<0.001
Regularly exercise			
Yes No	81 (18.8%) 137 (31.7%)	56 (13%) 158 (36.6%)	0.009
**Cancer awareness**			
Yes No	214 (49.5%) 4 (0.9%)	174 (40.3%) 40 (9.3%)	<0.001
**Contraceptive use**			
Hormonal Not-hormonal Don't use	55 (12.7%) 31 (7.2%) 132 (30.6%)	94 (21.8%) 32 (7.4%) 88 (20.4%)	<0.001
**Age of started menstruation**			
≤10 years old 11–14 years old ≤ 15 years old	55 (12.7%) 31 (7.2%) 132 (30.6%)	94 (21.7%) 32 (7.4%) 88 (20.4%)	<0.001

*Values are expressed as frequency (%, from the total) or mean ± SD*.

*Values of p are obtained by the Mann-Whitney test for the parametric variables or X^2^ for non-parametric variables*.

* *SR = Saudi Riyal (1 SR equals 0.27 American dollar)*.

Table 2 records the dietary habits of all participants regarding the main food groups. Fruits and vegetables (measured in servings per day), dairy products (servings per day), red and processed meat consumption (servings per day), and legumes (servings per week) showed significant results (*p* < 0.001) between the two groups. However, no differences (*p* > 0.05) between the two groups were found regarding the number of meals consumed per day, poultry intake, and whole wheat vs. white bread.

**Table 2:** Dietary habits for the main food groups between the case and control participants.

**Parameter**	**Control (*n* = 218)**	**Case (*n* = 214)**	***p*-value**
**Meals/day**			
<2 >3–5	57 (26.1%) 161 (73.9%)	70 (32.7%) 144 (67.3 %)	0.134
**Fruits and vegetables** **(servings/day)**			
<1 1–2 3–5 >5	24 (11%) 150 (68.8%) 42 (19.3%) 2 (0.9%)	38 (17.8%) 158 (73.8%) 14 (6.5%) 4 (1.9%)	<0.001
**Dairy products (servings/day)**			
<1 1–2 3–5 >5	4 (1.8%) 193 (88.5%) 19 (8.7%) 2 (0.9%)	58 (27.1 %) 134 (62.6%) 16 (7.5%) 6 (2.8%)	<0.001
**Red and processed meat (servings/day)**			
<1 1–2 3–5 >5	14 (6.4%) 152 (69.7%) 35 (16.1%) 17 (7.8%)	16 (7.5%) 94 (43.9%) 48 (22.4%) 56 (26.2%)	<0.001
**Poultry** **(servings/day)**			
<1 1–2 3–5 >5	24 (11%) 147 (67.4%) 40 (18.3%) 7 (3.2%)	28 (13.1%) 158 (73.8%) 26 (12.1%) 2 (0.9%)	0.093
**Legumes (servings/week)**			
<1 1–2 3–5 >5	10 (4.6%) 171 (78.4%) 33 (15.1%) 4 (1.8%)	40 (18.7%) 118 (55.1%) 48 (22.4%) 8 (3.7%)	<0.001
**Bread type**			
White Brown	103 (47.2%) 115 (52.8%)	110 (51.4%) 104 (48.6%)	0.388

*Values are expressed as frequency (%)*.

*Values of p are obtained by X^2^ test*.

Table 3 represents dietary habits by using the second group of common food items and drinks in the study groups. Results showed that there was a difference (*p* < 0.05) between the groups based on consumption of fish and seafood (measured in servings per week), leafy vegetables (servings per week), and olive oil (servings per week). Data measuring the consumption of black tea (cups per day) and coffee (cups per day) also showed a difference (*p* < 0.05) between the case and control groups. Only 8.3% of the control group ate <1 serving of fish and seafood every week when compared to 29.9% of the case group. Around 3.7% of the case group consumed <1 serving per week of green leafy vegetables, while only 1.4% from the control group ate <1 serving per week. Participants in the control group consumed olive oil (*p* < 0.001) more than the case group; 9.6% of the control group consumed <1 serving of olive oil per week, while 26.2% of the cancer cases consumed <1 serving of olive oil per week. About 56 and 36.4% of the control group and the case group, respectively, consumed 1–2 servings of olive oil per week. Participants with breast cancer tended to drink less black tea (in cups per day) as compared to healthy participants (*p* < 0.009): 18.7% of the participants with breast cancer consumed <1 cup of tea per day, while 10.1% of the healthy subjects consumed the same amount of tea daily. Subjects from the control group also consumed more coffee: only 9 control subjects (4.1%) drank <1 cup per day as compared to 48 subjects (22.4%) with breast cancer.

**Table 3:** Dietary habits of the study groups related to common foods and drinks.

**Parameter**	**Control (*n* = 218)**	**Case (*n* = 214)**	***p*-value**
**Fish and seafood** **(servings/week)**			
<1 1–2 3–5 >5	18 (8.3%) 153 (70.2%) 44 (20.2%) 3 (1.4%)	64 (29.9%) 133 (62.1%) 13 (6.1%) 4 (1.9%)	<0.001
**Leafy vegetables (servings/week)**			
<1 1–2 3–5 >5	3 (1.4%) 140 (64.2%) 53 (24.3%) 22 (10.1%)	8 (3.7%) 126 (58.9%) 32 (15%) 48 (22.4%)	<0.001
**Olive oil (servings/week)**			
<1 1–2 3–5 >5	21 (9.6%) 122 (56%) 40 (18.3%) 35 (16.1%)	56 (26.2%) 78 (36.4%) 40 (18.7%) 40 (18.7%)	<0.001
**Black tea (cup/day)**			
<1 1–2 3–5 >5	22 (10.1%) 45 (20.6%) 19 (8.7%) 132 (60.6%)	40 (18.7%) 48 (22.4%) 7 (3.3%) 119 (55.6%)	0.009
**Coffee (cup/day)**			
<1 1–2 3–5 >5	9 (4.1%) 63 (28.9%) 29 (13.3%) 117 (53.7%)	48 (22.4%) 86 (40.2%) 11 (5.1%) 69 (32.3%)	<0.001

*Values are expressed as frequency (%)*.

*Values of p are obtained by X^2^ test*.

Potential dietary habits related to breast cancer incidence are shown in Table 4. The consumption of 1–2 servings of dairy products per day was shown to be preventive against breast cancer (OR = 0.178, 95% CI = 0.037–0.859, *p* = 0.032), as was the consumption of 3–5 servings of dairy products daily (OR = 0.038, 95% CI = 0.004–0.372, *p* = 0.005). Results also showed a preventive effect of consuming 1–2 servings of legumes per week (OR = 0.043, 95% CI = 0.01–0.191, *p* < 0.001), 3–5 servings of fruits and vegetables per day (OR = 0.161, 95% CI = 0.043–0.605, *p* = 0.007), 1–2 servings of fish and sea food per week (OR = 0.211, 95% CI = 0.82–0.545, *p* = 0.001), and 3–5 servings of fish and sea food per week (OR = 0.072, 95% CI = 0.202–0.265, *p* < 0.001). Drinking 1–2 cups of tea (OR = 0.06, 95% CI = 0.01–0.371, *p* = 0.002) or 3–5 cups of tea daily was shown to reduce the incidence of breast cancer (OR = 0.083, 95% CI = 0.009–0.395, *p* = 0.003). The daily intake of 1–2 cups of coffee (OR = 0.159, 95% CI = 0.031–0.812, *p* = 0.027), 3–5 cups of coffee (OR = 0.083, 95% CI = 0.013–0.544, *p* = 0.009), or even more than 5 cups of coffee per day (OR = 0.144, 95% CI = 0.028–0.736, *p* = 0.02) had shown to reduce the incidence of breast cancer.

**Table 4:** Potential dietary habits as predictors for breast cancer.

***p*-value**	**95% CI**	**OR**	**β**	**Independent variable**
**Dairy products (servings/day)**				
<1 1–2 3–5 >5	0 −1.727 −3.269 −0.301	1 0.178 0.038 0.740	0.037–0.859 0.004–0.372 0.02–27.748	0.032 0.005 0.871
**Legumes (servings/week)**				
<1 1–2 3–5 >5	0 −3.135 −1.038 −1.146	1 0.043 0.354 0.318	0.01–0.191 0.074–1.694 0.031–3.314	<0.001 0.194 0.338
**Fruits and vegetables (servings/day)**				
<1 1–2 3–5 >5	0 −0.519 −1.837 −0.460	1 0.595 0.161 0.631	0.228–1.55 0.043–0.605 0.032–12.401	0.288 0.007 0.762
**Leafy vegetables (servings/week)**				
<1 1–2 3–5 >5	0 −0.899 −1.450 1.112	1 0.407 0.234 3.040	0.006–27.9 0.003–16.867 0.041–223.42	0.677 0.506 0.612
**Fish and seafood (servings/week)**				
<1 1–2 3–5 >5	0 −1.557 −2.631 −1.267	1 0.211 0.072 0.282	0.82–0.545 0.202–0.265 0.015–5.194	0.001 <0.001 0.394
**Red and processed meat (servings/day)**				
<1 1–2 3–5 >5	0 −0.216 0.383 1.001	1 0.806 1.466 2.72	0.137–4.746 0.222–9.695 0.405–18.280	0.811 0.691 0.303
**Olive oil (servings/week)**				
<1 1–2 3–5 >5	0 −0.405 0.763 0.422	1 0.667 2.145 1.524	0.218–2.042 0.574–8.014 0.426–5.454	0.478 0.256 0.517
**Black tea (cups/day)**				
<1 1–2 3–5 >5	0 −2.811 −2.820 −1.062	1 0.06 0.06 0.346	0.01–0.371 0.009–0.395 0.106–1.131	0.002 0.003 0.079
**Coffee (cups/day)**				
<1 1–2 3–5 >5	0 −1.842 −2.487 −1.940	1 0.159 0.083 0.144	0.031–0.812 0.013–0.544 0.028–0.736	0.027 0.009 0.02

*The reference is the control group*.

*All variables were adjusted for all other confounders*.

*β, beta coefficient; CI, confidence interval; OR, odds ratio*.

## Discussion

In Saudi Arabia, the rate of breast cancer ranges from three to eight confirmed cases for every 1,000 patients. Breast cancer accounts for 14.8% of all cancers reported among Saudi nationals and about 29% of cancers among women of all ages (2). It is critically important to assess the dietary factors associated with breast cancer in the Makkah region. This study showed that consuming healthy foods that include black tea, coffee, fruits and vegetables, fish and seafood, legumes, and dairy products can be preventive factors against breast cancer.

Our study results showed that consuming one to five servings of dairy products daily, a major source of vitamin D for Saudi women, had up to a 96% preventive effect against breast cancer. Dairy products in Saudi Arabia are fortified with vitamin D, which has been shown to reduce the risk of breast cancer by multiple mechanisms: either by promoting cell differentiation, decreasing cancer cell growth, stimulating cell death (apoptosis), or by reducing the formation of blood vessels in the tumor (angiogenesis) (15, 16). A recent review study supported our study findings that vitamin D had an inverse relationship with breast cancer (17). A descriptive study conducted in Saudi Arabia by AlFaris et al. (18), in contrast, demonstrated the results of the intake of vitamin D on breast cancer incidence that did not support our findings. This can be explained by vitamin D deficiency among both the control group and the cancer group in the previous study. The study also showed a relationship between symptoms of vitamin D deficiency and breast density, where women with mild to moderate breast density appeared to develop more deficiency symptoms.

Dairy products are also a good source of calcium, which plays a role in reducing the risk of breast cancer. Although the exact mechanism is still unclear, a meta-analysis of eleven studies by Hidayat et al. (19) showed a connection between calcium intake and breast cancer. The ability of calcium to regulate the cells' apoptosis, proliferation, and differentiation makes it a significant preventive factor. Both calcium and vitamin D were found to have anticarcinogenic effects in a review study by Cui (20). However, a recent meta-analysis published in 2019 by Chen et al. on the relationship between breast cancer and milk/yogurt intake did not support our results (21). This discrepancy may be due to the number of servings consumed by the subjects of Chen et al.'s study (21) and the amount of vitamin D added to the dairy products. Some studies have indicated that environmental pollutants, growth factors, and the amount and type of fat in milk can raise the risk of breast cancer. Another relevant question is whether cows that produce the milk were given bovine growth hormone, which results in an increase in the insulin-like factor-1 in milk and therefore might cause malignant cells to proliferate (21).

Conjugated linoleic acid (CLA) in dairy products is considered a chemoprotective agent. CLA also has antioxidant and anti-inflammatory effects that can decrease the risk of developing breast cancer (22). Research on the mechanism of CLA in reducing the risk of developing breast cancer is remarkably diverse, as described in a meta-analysis conducted by Zhou et al. (23). Another study by McCann et al. (24) did not support a clear correlation between CLA consumption and the development of breast cancer in pre- and postmenopausal women. They explained their result as being due to the high level of CLA consumption necessary for a preventive effect, as compared to the relatively low consumption by a typical test subject. Aro et al. (25) in their study found that breast cancer risk was higher in postmenopausal women who consumed a low amount of CLA from dairy products, but not in premenopausal women who consumed the same amount of CLA.

The study results also showed that the consumption of one to two servings of legumes per week had a negative association with breast cancer incidence. Various phytochemicals in legumes have an inhibitory effect on cell proliferation. Xu and Chang (26) comprehensively studied the effect of antioxidants and phytochemicals from different common types of legumes against nine different types of cancer, such as breast cancer, and they found that legumes are an outstanding source of natural antioxidants for the reduction of oxidative stress and cancer prevention. The fiber content in legumes was additionally found to inhibit the enterohepatic circulation of estrogen, leading to the reduction of the circulating estrogen level and resulting in a reduction of breast cancer risk (27). Fiber is also associated with decreasing cell mutation by binding with bile acid, which is thought to promote cell proliferation (28).

Fruits and vegetables are rich sources of fibers that are known to protect against breast cancer. Women who have a daily intake of three to five servings of fruits and vegetables are 83.9% less likely to develop breast cancer. An important study by Farvid et al. (29) concluded that high fiber intake from fruits and vegetables reduces the risk of developing breast cancer in women. Antioxidants in fruits and vegetables have been shown to neutralize free radicals and prevent DNA damage that might lead to cancer (29). Additionally, Naja et al. (28) concluded that the consumption of both fruits and vegetables reduces breast cancer risk. Many mechanisms could explain the preventive effect of fruit and vegetable consumption. Fiber content may bind to estrogen, thereby inhibiting the estrogen enterohepatic reabsorption. Antioxidants in fruits and vegetables also reduce oxidative stress and inflammation by protecting the DNA from damage and by inducing detoxifying enzymes. Vitamin C, found especially in citrus fruits, has been shown to benefit the immune system, while vitamin E and carotenoids have been found to have chemopreventive effects (29).

Women in the Makkah region consume seafood and fish regularly, especially on holidays. Our study indicates that consuming up to 5 servings of fish and seafood weekly reduces the risk of breast cancer by 78.9–92.8%. The positive effect of fish consumption appeared to be limited to certain common types of cancer, such as breast cancer (30). Engeset et al. (31), however, found no evidence of an inverse correlation between overall fish intake and the risk of breast cancer. Fish is a known source of omega 3 fatty acids, or polyunsaturated fatty acids (PUFAs), which can reduce the risk of breast cancer (32). Their study showed that PUFAs inhibited the epidermal growth factor receptor, which in turn reduced the proliferation of breast cancer.

Our study found that the consumption of coffee and black tea had a negative relationship to the development of breast cancer. In black tea, several antioxidant compounds and chemoprotective components are well known; catechins, particularly gallate epigallocatechin, exerted important antioxidant properties by decreasing the number of reactive oxygen species (33). Other coffee components, such as cafestol and kahweol, are known to have antioxidant and anticarcinogenic effects. Kahweol restricts the proliferation of the breast cells and causes apoptosis; it also increases the synthesis of reactive oxygen species to produce cytotoxicity (34). Other proposed mechanisms by which coffee may reduce the risk of breast cancer are though to be associated with induction of apoptosis and reducing inflammatory markers in the circulation (33). Yang et al. (35) explained the anticancerogenic mechanisms of black tea: its antioxidant effect protects the cell and DNA from being damaged by the free radicals. The phenolic compounds in coffee are known to have antioxidant, antimutagenic, and anticarcinogenic effects against several forms of cancer. A follow-up study by Ganmaa et al. (36) found a weak inverse link between caffeine consumption and the risk of postmenopausal breast cancer. Another article from Saudi Arabia supported our findings that caffeine content in coffee and tea provides effective prevention of breast tumor growth and/or recurrence (37).

A systematic review and meta-analysis published in 2015 indicated that the increased consumption of total saturated fat positively affected breast cancer incidence (38). Moreover, high consumption of meat has also been shown to increase the risk of developing breast cancer (39). However, study results did not show a remarkable effect of high fat intake from meat and processed meat and the risk of breast cancer. It is noteworthy to perform further studies regarding this point considering the different types of meat and processed meat consumed locally.

This study is limited by the semiquantitative measurement of food intake, which is a common problem with studies that use food frequency questionnaires and recall bias. This study was also limited by the regional sample collection, relatively small sample size, and recruitment of exclusively postmenopausal women for both case and control groups. Furthermore, it is noteworthy to perform further studies about the association between the adherence to the Mediterranean diet and breast cancer incidence and/or mortality rate.

## Conclusions

Our study in the Makkah region concluded that the consumption of fish and seafood, fruits and vegetables, legumes, coffee, black tea, and dairy products can have preventive effects against breast cancer. Dietary factors of the Mediterranean diet that did not show a significant effect on breast cancer incidence included olive oil, whole wheat, and leafy vegetables. It is recommended to perform additional studies using cohort study design in various regions of Saudi Arabia and with more participants.

## Data Availability Statement

The raw data supporting the conclusions of this article will be made available by the authors, without undue reservation.

## Ethics Statement

The studies involving human participants were reviewed and approved by Institutional Review Board of Umm Al-Qura University. The patients/participants provided their written informed consent to participate in this study.

## Author Contributions

FA conceived and designed the study. DH, AQ, KG, WA, AAA, AFA, HAA, and MG conducted research, provided research materials, and collected and organized data. HMA, MA, MH, AYA, SM, MQ, and WB analyzed and interpreted data. All authors wrote the initial and final drafts of the article and critically reviewed and approved the final draft of the manuscript.

## Funding

Taif University Researchers Supporting Project number (TURSP-2020/350), Taif University, Saudi Arabia.

## Conflict of Interest

The authors declare that the research was conducted in the absence of any commercial or financial relationships that could be construed as a potential conflict of interest.

## Publisher's Note

All claims expressed in this article are solely those of the authors and do not necessarily represent those of their affiliated organizations, or those of the publisher, the editors and the reviewers. Any product that may be evaluated in this article, or claim that may be made by its manufacturer, is not guaranteed or endorsed by the publisher.
