# Corrigendum: Consumption of Food Components of the Mediterranean Diet Decreases the Risk of Breast Cancer in the Makkah Region, Saudi Arabia: A Case-Control Study

**DOI:** 10.3389/fnut.2022.924278

**Published:** 2022-05-09

**Authors:** Firas S. Azzeh, Deena M. Hasanain, Alaa H. Qadhi, Khloud J. Ghafouri, Wedad F. Azhar, Mazen M. Ghaith, Abdullah F. Aldairi, Hussain A. Almasmoum, Hamza M. Assaggaf, Maha H. Alhussain, Ahmad A. Alghamdi, Mahmoud M. Habibullah, Waleed M. Bawazir, Sofyan S. Maghaydah, Maysoun S. Qutob, Awfa Y. Alazzeh

**Affiliations:** ^1^Clinical Nutrition Department, Faculty of Applied Medical Sciences, Umm Al-Qura University, Makkah, Saudi Arabia; ^2^Clinical Nutrition and Dietetics Department, Faculty of Pharmacy, Applied Science Private University, Amman, Jordan; ^3^Clinical Nutrition Department, King Abdullah Medical City in Holy Capital (KAMC-HC), Makkah, Saudi Arabia; ^4^Department of Laboratory Medicine, Faculty of Applied Medical Sciences, Umm Al-Qura University, Makkah, Saudi Arabia; ^5^Department of Food Science and Nutrition, College of Food and Agriculture Sciences, King Saud University, Riyadh, Saudi Arabia; ^6^Department of Clinical Laboratories Sciences, College of Applied Medical Sciences, Taif University, Taif, Saudi Arabia; ^7^Medical Laboratory Technology Department, Faculty of Applied Medical Sciences, Jazan University, Jazan, Saudi Arabia; ^8^Medical Research Center, Jazan University, Jazan, Saudi Arabia; ^9^Department of Medical Laboratory Technology, Faculty of Applied Medical Sciences, King Abdulaziz University, Jeddah, Saudi Arabia; ^10^Hematology Research Unit, King Fahd Medical Research Center, King Abdulaziz University, Jeddah, Saudi Arabia; ^11^Department of Clinical Nutrition, College of Applied Medical Sciences, University of Ha'il, Ha'il, Saudi Arabia

**Keywords:** breast cancer, postmenopausal, Mediterranean diet, dietary habits, nutrition

In the original article there was an error in affiliation 10 as published. Instead of Makkah the city should be “Jeddah.”

The authors apologize for this error and state that this does not change the scientific conclusions of the article in any way. The original article has been updated.

## Publisher's Note

All claims expressed in this article are solely those of the authors and do not necessarily represent those of their affiliated organizations, or those of the publisher, the editors and the reviewers. Any product that may be evaluated in this article, or claim that may be made by its manufacturer, is not guaranteed or endorsed by the publisher.

